# Prognostic values of EORTC QLQ-C30 and QLQ-HCC18 index-scores in patients with hepatocellular carcinoma – clinical application of health-related quality-of-life data

**DOI:** 10.1186/s12885-016-2995-5

**Published:** 2017-01-04

**Authors:** Leung Li, Frankie KF Mo, Stephen L Chan, Edwin P Hui, Nelson SL Tang, Jane Koh, Linda KS Leung, Annette NY Poon, Joyce Hui, Cheuk M Chu, Kit F Lee, Brigette BY Ma, Paul BS Lai, Anthony TC Chan, Simon CH Yu, Winnie Yeo

**Affiliations:** 1 Comprehensive Cancer Trials Unit, Department of Clinical Oncology, State Key Laboratory in Oncology in South China, Prince of Wales Hospital, Faculty of Medicine, The Chinese University of Hong Kong, Shatin, Hong Kong SAR; 2 Department of Diagnostic and Interventional Radiology, Prince of Wales Hospital, Shatin, Hong Kong SAR; 3 Department of Surgery, Prince of Wales Hospital, Shatin, Hong Kong SAR; 4 Department of Chemical Pathology, Li Ka Shing Institute of Health Sciences, Faculty of Medicine, The Chinese University of Hong Kong, Shatin, Hong Kong SAR

**Keywords:** Health-related quality-of-life, QLQ-HCC18, QLQ-C30, Index-score, Prognosis, Overall survival, Hepatocellular carcinoma, Liver cancer

## Abstract

**Background:**

Health-related quality-of-life (HRQOL) assessment with EORTC QLQ-C30 was prognostic for overall survival (OS) in patients with advance-stage hepatocellular carcinoma (HCC), but no data existed for early-stage patients. The HCC-specific QLQ-HCC18 has not been evaluated for prognostic value in HCC patients. Utilization of raw HRQOL data in clinical setting has been impractical and non-meaningful. Therefore we developed index scores of QLQ-C30 and QLQ-HCC18 in an attempt to enable clinical utilization of these HRQOL measurements. This study investigates the prognostic significance of QLQ-C30, QLQ-HCC18 and C30/HCC18 index-scores in patients with newly diagnosed HCC which encompasses all stages.

**Methods:**

From 2007–2011, 517 patients were prospectively recruited. HRQOL was assessed at diagnosis using QLQ-C30 and QLQ-HCC18; C30 and HCC18 index-scores were calculated from raw HRQOL data. Cox regression was performed using continuous, dichotomized QLQ-C30 and QLQ-HCC18 variables, or index-scores, together with clinical factors to identify independent factors for OS. Various multivariate models were validated with c-index and bootstrapping for 1000 replications.

**Results:**

Four hundred and seventy two patients had complete HRQOL data. Their median OS was 8.6 months. In multivariate analysis, independent prognostic HRQOL variables for OS were QLQ-C30 pain (HR 1.346 [1.092–1.661], *p* = 0.0055), QLQ-C30 physical functioning (HR 0.652 [0.495–0.860], *p* = 0.0024); QLQ-HCC18 pain (HR 1.382 [1.089–1.754], *p* = 0.0077) and QLQ-HCC18 fatigue (HR 1.441 [1.132–1.833], *p* = 0.0030). C30 index-score (HR 2.143 [1.616–2.841], *p* < 0.0001) and HCC18 index-score (HR 1.957 [1.411–2.715], *p* < 0.0001) were highly significant factors for OS. The median OS of patients with C30 index-score of 0–20, 21–40, 41–60, 61–100 were 16.4, 7.3, 3.1, 1.8 months respectively (*p* < 0.0001); while for HCC18 index-score: 16.4, 6.0, 2.8, 1.8 months respectively (*p* < 0.0001). All the multivariate models were validated, with mean optimism <0.01. The bootstrap validated c-index was 0.78.

**Conclusions:**

QLQ-C30 and QLQ-HCC18 were prognostic for OS in patients with newly diagnosed HCC irrespective of stage. Both C30 and HCC18 index-scores were highly significant prognostic factors for OS in newly diagnosed HCC patients. Index-scoring provides an effective way to summarize, analyze and interpret raw HRQOL data, and renders QLQ-C30 and QLQ-HCC18 meaningful and communicable in clinical practice. Index-scores could potentially serve as a standardized tool for future HRQOL research.

## Background

Three studies have shown health-related quality-of-life (HRQOL) being prognostic for overall survival (OS) in patients with advance-stage hepatocellular carcinoma (HCC) [1–3]. These used general cancer HRQOL measurement tools, namely the European Organization for Research and Treatment of Cancer (EORTC) QLQ-C30 [4] and Spitzer QOL index [5]. On the other hand, one negative study recruited both early- and advance-stage HCC patients and used another general cancer HRQOL measurement, Functional Assessment of Cancer Therapy – General (FACT-G) [6, 7]. To date, there has been no study evaluating the prognostic value of QLQ-C30 for patients with newly diagnosed HCC which encompasses all stages.

Patients with HCC often suffer from chronic liver disease. In Asia, this is mainly due to chronic hepatitis B virus (HBV) infection [8–10]. Therefore liver-specific HRQOL measurement could be more relevant for these patients. EORTC QLQ-HCC18 [11] is a specific HRQOL module which addresses QOL issues specific for patients with primary liver cancer. It has been validated in Asian HCC patients [12, 13] and many scales of QLQ-HCC18 have been reported to enable the identification of patients with different clinical conditions. However, the prognostic value of EORTC QLQ-HCC18 in HCC patients has not been evaluated.

So far it has been a common practice to analyze raw HRQOL data as a collection of continuous variables, and various HRQOL factors have been proven to be prognostic for survival in various malignancies. Despite the wide utilization of EORTC QLQ-C30, there has been no domain/item identified to be consistently prognostic [14]. Difficulties in HRQOL research were well recognized: multi-collinearity among numerous raw HRQOL data causing multivariate analysis model instability, overfitting of variables leading to excessive multiple comparisons and type I error [14, 15], and lack of means to meaningfully translate raw HRQOL data into clinical use. Diouf et al. dichotomized all HRQOL data at a universal cut-off at 50 for analysis. This addressed the issues of multi-collinearity and overfitting and provided a way to interpret HRQOL data by clinicians [3]. A separate analysis was performed to determine the true cut-off for various domains/items, and these cut-offs have been considered to be potentially population-specific [16].

In an attempt to determine a generalizable way to analyze and interpret HRQOL data while minimizing multi-collinearity and over-fitting, we derived two index-scores, namely the C30 and HCC18 index-scores, to represent all domains and items within the EORTC QLQ-C30 and QLQ-HCC18 respectively.

The objectives of this study are: (1) to evaluate the prognostic value of QLQ-C30 in a prospective cohort of newly diagnosed patient with HCC which encompasses all stages; (2) to investigate the prognostic significance of the liver-specific QLQ-HCC18 in this cohort; and (3) to evaluate the prognostic significance of C30 and HCC18 index-scores.

## Methods

From January 2007 to December 2011, all patients with newly diagnosed HCC presented to the multidisciplinary hepatoma clinic of Prince of Wales Hospital were considered for recruitment. The study was approved by the Joint Chinese University of Hong Kong-New Territories East Cluster Clinical Research Ethics Committee.

Eligibility criteria included: adult patients with newly diagnosed and treatment-naïve HCC; the diagnosis of HCC as confirmed by either histology, the combination of radiological and biochemical findings (space-occupying lesion in the liver with raised α-fetoprotein (AFP ≥ 400ug/L), or 2 typical radiological findings with ultrasonography, triphasic computed tomography, angiography or magnetic resonance imaging; ability to read and comprehend Chinese was a pre-requisite. Patients were excluded if they had history of malignancy, encephalopathy or cognitive impairment.

### Treatment

After confirmation of diagnosis and stage, patients were offered appropriate treatment as clinically indicated. Treatment options included surgical resection, local ablative therapies – radiofrequency ablation (RFA) or percutaneous ethanol injection (PEI), transarterial therapies - transarterial chemo-embolisation (TACE) or transarterial injection of lipiodol-ethanol mixture (LEM), systemic therapies – sorafenib, chemotherapy, clinical trials and best supportive care (BSC) alone.

### HRQOL assessment

Consented patients would complete two HRQOL questionnaires: the EORTC QLQ-C30 and QLQ-HCC18, at their baseline visit before treatment commencement.

### EORTC QLQ-C30

The Chinese version of QLQ-C30 was used [4]. It is a cancer-specific 30-item questionnaire composed of multiple items that reflect the multidimensionality of HRQOL construct, presented in multiple-point Likert scales. These items are grouped into 9 domains and 6 single items. It incorporates 5 functional domains (physical, role, cognitive, emotional and social), 3 symptom domains (fatigue, pain, nausea and vomiting) and a global health and QOL domain. The remaining 6 single items assess additional 5 symptoms commonly reported by cancer patients (dyspnea, appetite loss, sleep disturbance, constipation and diarrhea) as well as perceived financial problem. All domains and scales were converted to scores ranging from 0 to 100 according to the scoring manual [17]. A higher score for a functional or global QOL scale represents a relatively higher/healthier level of functioning or global QOL, whereas a higher score for a symptom/problem scale represents a more severe symptom/problem.

### EORTC QLQ-HCC18

The Chinese version of EORTC QLQ-HCC18 [11] includes 18 multi-item scales. These items are grouped into 6 domains namely fatigue, body image, jaundice, nutrition, pain and fever. Two remaining single items address abdominal swelling and sex life. All scales were grouped and converted to score 0 to 100 according to the scoring manual; a higher score represents a more severe symptom or problem.

### C30 and HCC18 index-scores

C30 and HCC18 index-scores were derived in order to have an overall representation of all domains/items in QLQ-C30 and QLQ-HCC18 respectively.

To calculate C30 index-score, individual functioning scale was subtracted by 100 to convert them into having the same meaning as symptom/problem scales. These 6 subtracted scores were subsequently summed with the 9 symptom/problem scales, and then divided by 15 (the total number of QLQ-C30 scales). A higher C30 index-score reflects a worse overall HRQOL. This is the mathematical formula:

$$ \begin{array}{l}\mathsf{C}\mathsf{30}\ \mathsf{index}\hbox{-} \mathsf{score}=\varSigma \left[\left(\mathsf{100}\hbox{-} \mathsf{Physical}\ \mathsf{functioning}\right),\Big(\mathsf{100}\hbox{-} \mathsf{Role}\ \mathsf{functioning}\right),\left(\mathsf{100}\hbox{-} \mathsf{Emotional}\ \mathsf{functioning}\right),\ \\ {}\left(\mathsf{100}\hbox{-} \mathsf{Cognitive}\ \mathsf{functioning}\right),\ \left(\mathsf{100}\hbox{-} \mathsf{Social}\ \mathsf{functioning}\right),\ \left(\mathsf{100}\hbox{-} \mathsf{global}\ \mathsf{Q}\mathsf{O}\mathsf{L}\right),\ \mathsf{scores}\ \mathsf{of}\ \mathsf{Fatigue},\ \\ {}\mathsf{Nausea}/\mathsf{vomiting},\ \mathsf{Pain},\ \mathsf{Dyspnoea},\ \mathsf{Insomnia},\ \mathsf{Appetite}\ \mathsf{loss},\ \mathsf{C}\mathsf{onstipation},\ \mathsf{Diarrhea},\ \mathsf{Financial}\ \mathsf{Diffculty}\Big] \div \mathsf{15}\end{array} $$

HCC18 index-score was defined as the sum of all 8 QLQ-HCC18 symptom/problem scales divided by 8 (the total number of QLQ-HCC18 scales). A higher HCC18 index-score reflects a worse overall HRQOL. This is the mathematical formula:

$$ \mathsf{H}\mathsf{C}\mathsf{C}\mathsf{18}\mathsf{index}{\textstyle \hbox{-}}\mathsf{score}=\varSigma \left(\mathsf{scoresofFatigue},\mathsf{BodyImage},\mathsf{Jaundice},\mathsf{Nutrition},\mathsf{Pain},\mathsf{Fever},\mathsf{Sexlife},\mathsf{Abdominaldistension}\right)\div \mathsf{8} $$

### Clinical factors and follow-up

Demographic, clinical and laboratory parameters were collected. All patients were followed up for treatment and monitoring until death or last contact.

### Statistical analysis

Standard descriptive analyses were performed to assess sample characteristics. OS was defined as the time from the date-of-consent to date-of-death. In the absence of death confirmation, survival time was censored at the date-of-last-seen. Survival estimation was performed by the Kaplan-Meier method, and compared using the log-rank test.

Only patients with complete HRQOL data were included in statistical analysis. EORTC QLQ-C30 and QLQ-HCC18 scales were included in the prognostic factor analysis as (i) continuous variables, (ii) dichotomized (≥50 or <50) variables, and (iii) index-scores. Univariate analysis was performed with baseline HRQOL scores and non-overlapping clinical variables to identify factors that influenced survival using Cox proportional-hazards regression model. A stepwise model building procedure was used for multivariate analysis, based on a significance value of 0.05 for both inclusion and exclusion of prognostic factors. For analyses involving continuous variables, higher scores (better function and worse symptoms/problems respectively) were compared to lower scores (worse function or better symptoms/problems); whereas for dichotomized data, symptom/problem domain/item scores of ≥50 (worse scores) were compared to <50 (better scores), while functional domain scores of <50 (worse scores) were compared to ≥50 (better scores).

Treatment options were grouped into curative-intent treatment (surgical/locoablative therapies), palliative-intent treatment (transarterial/systemic therapies) or BSC.

Performance of the final multivariate models were assessed and compared by Harrell’s concordance-index (c-index) [18]. The c-index estimates the probability of concordance between predicted and observed responses. A value of 1.0 indicates perfect separation of patients with different outcomes, and a value of 0.5 indicates no predictive discrimination. Internal validation was carried out by comparing the c-index of each model with the c-indexes of 1000 bootstrap replications to obtain optimisms, which were averaged and bootstrap-corrected performance was estimated.

The statistical analyses were performed using SAS version 9.3 software package. A p-value of less than 0.05 was considered significant. The c-index and 95% confidence intervals (CI) of the different models were calculated by using the SAS macro program.

## Results

### Patient characteristics

Among the 517 patients who consented, 472 (91%) had complete HRQOL data and were included for analysis. Table 1 listed the clinical characteristics of these patients. The median age at diagnosis was 60, the majority were male (89%). Most patients had Eastern Cooperative Oncology Group (ECOG) performance status 0–1 (94%). HBV infection was present in 82%, while hepatitis C in 6%. Fifty nine percent had cirrhosis and 68% was of Child-Pugh class A. Eighteen percent of patients received first-line curative intent treatment, the rest received palliative treatment (44%) or BSC (38%).

**Table 1:** Baseline characteristics and univariate Cox regression analyses of overall survival for patients with complete HRQOL data (*n* = 472)

	*n*	%	HR	95% C.I.	*p*-value
Demographics/clinical					
Age < = 65	328	69	0.909	0.730–1.131	0.3898
Male gender	419	89	1.299	0.919–1.835	0.1381
ECOG ≥ 2	29	6	2.885	1.927–4.317	<0.0001
Laboratory					
Hemoglobin <10g/dL	27	6	1.294	0.840–1.995	0.2424
White cell count >10×10^9^/L	64	14	2.507	1.885–3.333	<0.0001
Platelet count < 100×10^9^/L	33	7	1.696	1.158–2.485	0.0067
International normalized ratio >1.4	36	8	1.410	0.945–2.104	0.0922
Creatinine ≥ ULN	67	14	1.115	0.833–1.491	0.4644
Bilirubin ≥ 20umol/l	239	51	1.974	1.594–2.445	<0.0001
Albumin ≤35g/l	182	39	2.186	1.765–2.708	<0.0001
Alanine aminotransferase >2xULN	81	17	1.567	1.201–2.044	0.0009
Alkaline phosphatase >2xULN	145	31	2.417	1.938–3.014	<0.0001
Underlying liver condition					
Hepatitis B surface antigen +	386	82	1.196	0.908–1.575	0.2029
Hepatitis C antibody +	30	6	0.641	0.403–1.019	0.0600
Ascites	278	59	0.894	0.721–1.107	0.3036
Cirrhosis (radiological)	122	26	2.237	1.775–2.820	<0.0001
Child-Pugh class					
A	319	68	1.000	-	-
B	130	28	2.187	1.740–2.750	<0.0001
C	23	4	4.360	2.774–6.854	<0.0001
Tumor characteristics					
α-fetoprotein ≥200 mg/ml	250	53	2.318	1.865–2.882	<0.0001
Tumor morphology					
Uninodular	122	26	1.000	-	-
Multinodular	143	30	2.097	1.509–2.914	<0.0001
Diffuse	207	44	4.360	3.027–3.822	<0.0001
Extrahepatic metastasis (nodal or distant)	108	23	3.003	2.360–3.822	<0.0001
Portal vein thrombosis	152	32	3.647	2.899–4.587	<0.0001
1^st^ line Treatment					
Curative	83	18	1.000	-	
Palliative or best supportive care alone	389	82	5.802	3.820–8.810	<0.0001
Surgical treatment	54	12	1.000	-	
Local ablative therapies	29	6	2.526	1.131–5.643	0.0238
Trans-arterial therapies	116	25	4.597	2.446–8.637	<0.0001
Systemic therapies	91	19	10.209	5.380–19.372	<0.0001
Best supportive care alone	182	38	15.624	8.442–28.914	<0.0001

*ECOG* Eastern Cooperative Oncology Group performance status, *ULN* upper limit of normal, *CI* confidence interval, *HR* hazard ratio

The median follow-up duration was 29.8 months (95% CI [26.8–32.8]), 377 patients had died. The median OS was 8.6 months (95% CI [7.3–10.2]).

Mean scores of QLQ-HCC18 and QLQ-C30 scales and mean HCC18 and C30 index-scores were listed in Table 2.

**Table 2:** Baseline HRQOL and univariate analyses using continuous, dichotomized variables, C30 and HCC18 index scores for patients with complete HRQOL data (*n* = 472)^a^

	Mean	±SD	Continuous variables		Dichotomized variables^a^	
			HR [95% CI]	p-value	HR [95% CI]	*p*-value
EORTC QLQ-C30						
Physical Functioning	72.27	23.74	0.432 [0.351–0.533]	<0.0001	2.026 [1.571–2.612]	<0.0001
Role Functioning	74.61	32.60	0.517 [0.443–0.603]	<0.0001	2.108 [1.645–2.702]	<0.0001
Emotional Functioning	70.67	25.48	0.777 [0.632–0.954]	0.0164	1.448 [1.100–1.908]	0.0084
Social Functioning	76.80	24.68	0.698 [0.571–0.853]	0.0004	1.611 [1.162–2.234]	0.0042
Cognitive Function	68.46	30.33	0.634 [0.531–0.756]	<0.0001	1.573 [1.233–2.007]	0.0003
Global Quality of Life	52.22	26.34	0.515 [0.417–0.635]	<0.0001	1.611 [1.299–1.998]	<0.0001
Fatigue	42.93	30.23	1.973 [1.657–2.349]	<0.0001	2.072 [1.672–2.568]	<0.0001
Nausea/Vomiting	11.26	21.41	2.050 [1.643–2.559]	<0.0001	2.308 [1.679–3.173]	<0.0001
Pain	32.87	31.97	1.865 [1.584–2.197]	<0.0001	2.108 [1.698–2.617]	<0.0001
Dyspnoea	29.73	31.46	1.396 [1.189–1.639]	<0.0001	1.666 [1.314–2.113]	<0.0001
Insomnia	41.88	36.41	1.344 [1.162–1.556]	<0.0001	1.415 [1.144–1.750]	0.0014
Appetite loss	32.34	35.88	1.923 [1.668–2.217]	<0.0001	2.360 [1.889–2.949]	<0.0001
Constipation	16.67	27.13	1.201 [0.999–1.444]	0.0512	1.368 [1.021–1.834]	0.0359
Diarrhea	16.45	26.87	1.520 [1.252–1.845]	<0.0001	1.666 [1.248–2.224]	0.0005
Financial difficulties	51.20	37.22	1.353 [1.169–1.566]	<0.0001	1.579 [1.276–1.954]	<0.0001
C30 index score	30.69	19.61	3.658 [2.726–4.909]	<0.0001	-	-
EORTC QLQ-HCC18						
Fatigue	35.23	25.86	2.381 [1.942–2.919]	<0.0001	2.484 [1.968–3.136]	<0.0001
Body Image	25.35	22.98	2.261 [1.819–2.811]	<0.0001	2.167 [1.718–2.733]	<0.0001
Jaundice	23.41	22.15	1.289 [1.024–1.623]	0.0307	1.271 [0.977–1.652]	0.0739
Nutrition	26.96	21.35	2.934 [2.317–3.716]	<0.0001	2.663 [2.026–3.502]	<0.0001
Pain	23.34	24.57	2.107 [1.717–2.587]	<0.0001	1.871 [1.465–2.391]	<0.0001
Fever	6.60	14.39	1.568 [1.123–2.187]	0.0081	1.697 [0.929–3.099]	0.0851
Sex life	28.74	34.76	1.213 [1.040–1.415]	0.0139	1.436 [1.128–1.828]	0.0033
Abdominal swelling	33.33	35.43	1.721 [1.486–1.994]	<0.0001	2.192 [1.752–2.743]	<0.0001
HCC18 index score	25.37	17.21	3.028 [2.340–3.919]	<0.0001	-	-

C30 index-score = ∑ [(100-Physical functioning), (100-Role functioning), (100-Emotional functioning), (100-Cognitive functioning), (100-Social functioning), (100-global QOL), scores of Fatigue, Nausea/vomiting, Pain, Dyspnoea, Insomnia, Appetite loss, Constipation, Diarrhea, Financial Diffculty] ÷ 15

HCC18 index-score = ∑ (scores of Fatigue, Body Image, Jaundice, Nutrition, Pain, Fever, Sex life, Abdominal distension) ÷ 8

^a^In dichotomization, worse (≥50 in symptoms/problem or <50 in functioning/global QOL) scores in QLQ-C30 were analyzed with respect to better scores; worse (≥50) scores in QLQ-HCC18 were analyzed with respect to better scores

*SD* standard deviation, *CI* confidence interval, *HR* hazard ratio, *HRQOL* health-related quality of life

### Univariate analysis of HRQOL and clinical factors

Tables 1 and 2 summarized the univariate Cox regression analyses for clinical variables, QLQ-C30 and QLQ-HCC18 scores, as well as C30 and HCC18 index-scores.

#### Univariate HRQOL analysis based on continuous variables

For QLQ-C30, higher (better) scores in all functioning (e.g. physical functioning, HR 0.432 [0.351–0.533]) or global domains were significantly associated with longer OS (*p* < 0.03); whereas higher (worse) scores in fatigue, nausea/vomiting, pain (HR 1.865 [1.584–2.197]), dyspnea, insomnia, appetite loss, diarrhea and financial difficulties were significantly associated with shorter OS (*p* < 0.01).

For QLQ-HCC18, higher (worse) scores in all 8 symptom/problem domains (e.g. fatigue HR 2.381 [1.942–2.919]) were significantly associated with shorter OS (*p* < 0.05).

#### Univariate QOL analysis based on dichotomization of scores

For QLQ-C30, scores ≥50 (better) in global QOL, physical, role, cognitive and social functioning and scores <50 (better) in all symptom/problem domains (e.g. financial difficulties HR 1.579 [1.276–1.954]) were significantly associated with longer OS (*p* < 0.05).

For QLQ-HCC18, scores ≥50 (worse) in fatigue (HR 2.484 [1.968–3.136]), body image, nutrition, pain, sex life, abdominal swelling domains were significantly associated with shorter OS (*p* < 0.01).

#### Univariate QOL analysis based on the newly derived index-scores

Higher C30 index-score (reflecting worse overall functions/symptoms/problems) was significantly associated with shorter OS (HR 3.658 [2.726–4.909] *p* < 0.0001).

Higher HCC18 index-score (reflecting worse overall symptoms/problems) was significantly associated with shorter OS (HR 3.028 [2.340–3.919] *p* < 0.0001).

### Multivariate analysis of HRQOL data with clinical factors

Table 3 shows the results of the multivariate Cox regression analyses involving HRQOL variables or index-scores identified in univariate regression with non-overlapping clinical factors.

**Table 3:** Multivariate analysis of HRQOL variables or index scores with significant clinical factors (*n* = 472)

	Continuous QOL variables			Dichotomized QOL variables			Index score		
	HR	95% CI	*p*-value	HR	95% CI	*p*-value	HR	95% CI	*p*-value
EORTC QLQ-C30									
Physical Functioning	0.652	0.495–0.860	0.0024	1.475	1.095–1.986	0.0106	-	-	-
Pain	1.346	1.092–1.661	0.0055	1.523	1.192–1.947	0.0008	-	-	-
Financial difficulties	-	-	-	1.331	1.059–1.673	0.0141	-	-	-
C30 Index score	-	-	-	-	-	-	2.143	1.616–2.841	<0.0001
Portal vein thrombosis	1.723	1.342–2.212	<0.0001	1.702	1.325–2.187	<0.0001	1.661	1.291–2.136	<0.0001
Tumor Morphology – Multinodular	1.604	1.147–2.243	0.0058	1.614	1.152–2.260	0.0054	1.719	1.229–2.403	0.0015
Tumor Morphology – Diffuse	2.449	1.763–3.401	<0.0001	2.556	1.841–3.550	<0.0001	2.636	1.902–3.651	<0.0001
Albumin ≤35g/l	1.442	1.125–1.848	0.0039	1.541	1.199–1.981	0.0007	1.641	1.311–2.055	<0.0001
Bilirubin ≥20umol/l	1.785	1.400–2.275	<0.0001	1.784	1.398–2.277	<0.0001	1.752	1.390–2.208	<0.0001
α-fetoprotein ≥200 ng/ml	1.830	1.439–2.328	<0.0001	1.878	1.476–2.389	<0.0001	1.749	1.380–2.218	<0.0001
Extrahepatic metastasis	1.696	1.303–2.209	<0.0001	1.753	1.342–2.288	<0.0001	1.805	1.386–2.351	<0.0001
Alkaline phosphatase >2xULN	1.456	1.145–1.852	0.0022	1.420	1.116–1.806	0.0043	1.472	1.159–1.870	0.0015
Creatinine ≥ ULN	1.538	1.129–2.094	0.0063	1.637	1.204–2.227	0.0017	1.712	1.263–2.322	0.0005
Ascites	1.327	1.015–1.736	0.0385	1.325	1.012–1.734	0.0408	-	-	-
EORTC QLQ-HCC18									
Fatigue	1.441	1.132–1.833	0.0030	1.805	1.411–2.310	<0.0001	-	-	-
Pain	1.382	1.089–1.754	0.0077	-	-	-	-	-	-
HCC18 index score	-	-	-	-	-	-	1.957	1.411–2.715	<0.0001
Portal vein thrombosis	1.701	1.320–2.191	<0.0001	1.672	1.295–2.160	<0.0001	1.688	1.312–2.172	<0.0001
Tumor Morphology – Multinodular	1.638	1.172–2.289	0.0038	1.709	1.223–2.388	0.0017	1.681	1.203–2.348	0.0024
Tumor Morphology – Diffuse	2.510	1.805–3.490	<0.0001	2.813	2.034–3.891	<0.0001	2.624	1.893–3.637	<0.0001
Albumin ≤35g/l	1.684	1.344–2.111	<0.0001	1.704	1.360–2.135	<0.0001	1.666	1.329–2.088	<0.0001
Bilirubin ≥20umol/l	1.687	1.333–2.134	<0.0001	1.662	1.316–2.100	<0.0001	1.659	1.312–2.098	<0.0001
α-fetoprotein ≥200 ng/ml	1.744	1.371–2.218	<0.0001	1.805	1.423–2.289	<0.0001	1.735	1.367–2.201	<0.0001
Extrahepatic metastasis	1.788	1.370–2.334	<0.0001	1.830	1.402–2.389	<0.0001	1.773	1.361–2.309	<0.0001
Alkaline phosphatase >2xULN	1.426	1.124–1.810	0.0035	1.341	1.054–1.705	0.0169	1.445	1.139–1.832	0.0024
Creatinine ≥ ULN	1.695	1.249–2.301	0.0007	1.600	1.181–2.167	0.0024	1.701	1.253–2.348	0.0007

*ULN* upper limit of normal, *CI* confidence interval, *HR* hazard ratio, *HRQOL* health-related quality of life

#### Multivariate Analysis of clinical factors

Multifocal or diffuse HCC, presence of extra-hepatic metastasis, portal vein thrombosis, hypoalbuminemia, hyperbilirubinemia, high AFP, alkaline phosphatase and creatinine were consistently significant clinical factors associated with shorter OS in all multivariate analyses.

#### Multivariate HRQOL analysis based on continuous variables

For QLQ-C30, higher (better) score in physical functioning (HR 0.652 [0.495–0.860], *p* = 0.0024) was significantly associated with longer OS and higher (worse) score in pain (HR 1.346 [1.092–1.661], *p* = 0.0055) was significantly associated with shorter OS.

For QLQ-HCC18, higher (worse) scores in fatigue (HR 1.441 [1.132–1.833], *p* = 0.0030) and pain (HR 1.382 [1.089–1.754], *p* = 0.0077) were significantly associated with shorter OS.

#### Multivariate QOL analysis based on dichotomization of scores

For QLQ-C30, scores ≥50 (worse) in pain (HR 1.523 [1.192–1.947], *p* = 0.0008), and financial difficulties (HR 1.331 [1.059–1.673], *p* = 0.0141) and score <50 (worse) in physical functioning (HR 1.475 [1.095–1.986], *p* = 0.0106) were significant independent factors for shorter OS.

For QLQ-HCC18, worse fatigue score (≥50) (HR 1.805 [1.411–2.310], *p* < 0.0001) was significantly associated with shorter OS.

**Fig. 1:**
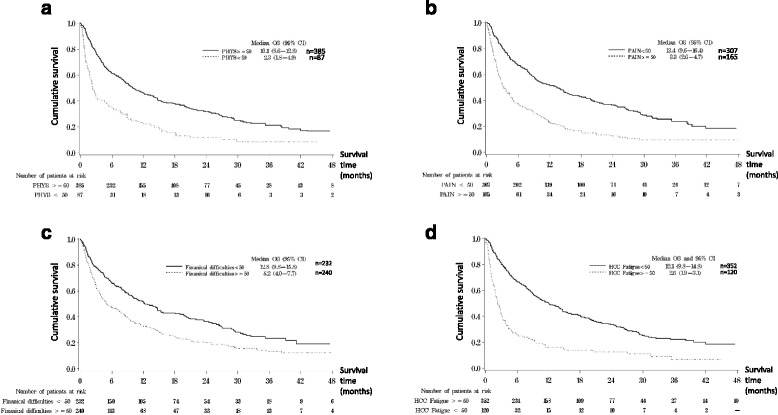
Overall survival curves for significant dichotomized HRQOL factors found in multivariate analysis. **a** QLQ-C30 Physical Functioning <50 vs ≥50. **b** QLQ-C30 Pain <50 vs ≥50. **c** QLQ-C30 Financial difficulties <50 vs ≥50. **d** QLQ-HCC18 Fatigue <50 vs ≥50. Phys: QLQ-C30 Physical functioning; HCC fatigue: QLQ-HCC18 Fatigue; OS: overall survival

Figure 1 shows the survival of patients with different scores in significant dichotomized HRQOL domains/items. The median OS for patients with better (≥50) and worse (<50) scores in QLQ-C30 physical functioning were 10.1 (95%CI 8.6–12.8) and 2.3 months (95% CI 1.9–4.9) respectively (*p* < 0.0001). The median OS for patients with better (<50) and worse (≥50) scores in QLQ-C30 pain were 13.4 (95% CI 9.6–16.4) and 3.3 months (95%CI 2.7–4.7) respectively (*p* < 0.0001), that of QLQ-C30 financial difficulties were 12.8 (95% CI 9.8–15.9) and 5.2 months (95% CI 4.0–7.8) (*p* < 0.0001) and QLQ-HCC18 fatigue were 12.1 (95% CI 9.8–14.8) and 2.6 months (95% CI 1.9–3.1) (*p* < 0.0001) respectively.

#### Multivariate QOL analysis based on the newly derived index-scores

In the multivariate analysis using C30 index-score with clinical factors, higher (worse) C30 index-score was a significant independent risk factor for shorter OS (HR 2.143 [1.616–2.841], *p* < 0.0001).

In the multivariate analysis using HCC18 index-score with clinical factors, higher (worse) HCC18 index-score was a significant independent risk factor for shorter OS (HR 1.957 [1.411–2.715], *p* < 0.0001).

**Fig. 2:**
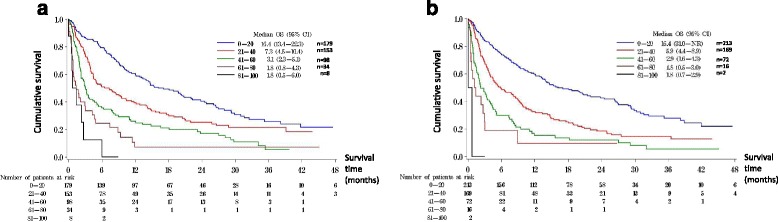
Overall survival curves according to stratified C30 and HCC18 index-scores. **a** Overall survival curves according to stratified C30 index-score. **b** Overall survival curves according to stratified HCC18 index-score

Figure 2 shows the OS plots for patients with stratified C30 and HCC18 index-scores respectively. Lower (better) C30 index-score ranges were associated with longer OS in a step-wise fashion (*p* < 0.0001); similarly, the lower (better) HCC18 index-score ranges, the longer the OS (*p* < 0.0001).

The median OS in patients with C30 index-score of 0–20 was 16.4 (95% CI 13.4–22.3) months, that for score 21–40 was 7.3 (95% CI 4.5–10.4) months, score 41–60 was 3.1 (95%CI 2.3–5.1) months, score 61–80 was 1.8 (95% CI 0.8–4.3) months, and score 81–100 was 1.8 (95% CI 0.5–6) months (*p* < 0.0001). The median OS of patients with HCC18 index-score of 0–20 was 16.4 (95% CI 31-not reached) months, that for score 21–40 was 5.9 (95%CI 4.4–8.9) months, score 41–60 was 2.9 (95% CI 1.6–4.3) months, score 61–80 was 1.8 (95% CI 0.5–3.0) months, and score 81–100 was 1.8 (95% CI 0.7–2.9) months (*p* < 0.0001) (Table 4).

**Table 4:** Overall survival data for patients stratified according to C30 and HCC18 index score range (*n* = 472)

	*N*	Median OS (M)	95% CI	Survival% at 6M	Survival% at 12M	Survival% at 24M	Survival% at 36M
C30 Index Score							
0–20	179	16.4	13.4–22.3	79.7	60.4	41.3	25.6
21–40	153	7.3	4.5–10.4	52.0	39.2	26.0	21.4
41–60	98	3.1	2.3–5.1	36.5	27.0	18.3	8.1
61–80	34	1.8	0.8–4.3	27.5	10.7	7.1	7.1
81–100	8	1.8	0.5–6.0	25.0	-	-	-
HCC18 Index Score							
0–20	213	16.4	31.0-NR	74.6	59.9	43.1	27.8
21–40	169	6.0	4.4–8.9	49.6	33.1	19.3	14.5
41–60	72	2.8	1.6–4.3	31.1	16.7	11.8	5.3
61–80	16	1.8	0.5–3.0	25.0	18.8	9.4	-
81–100	2	1.8	0.7–2.9	-	-	-	-

*M* month (s), *CI*, confidence interval, *NR* not reached

### Internal validation of the multivariate cox proportional hazard models

C-index of original dataset and mean c-index of 1000 bootstrap samples for multivariate model are described below. Using QLQ-C30 as continuous variables, the corresponding values were 0.7872 (95% CI 0.7648–0.8905) and 0.7891 (95% CI 0.7678–0.8111) respectively. Using QLQ-C30 as dichotomized variables, the values were 0.7842 (95% CI 0.7617–0.8066) and 0.7878 (95% CI 0.7660–0.8103) respectively. When assessed using C30 index score, the values were 0.7817 (95%CI 0.7591–0.8043) and 0.7840 (95% CI 0.7626–0.8066) respectively. Using QLQ-HCC18 as continuous variables, the values were 0.7810 (95% CI 0.7588–0.8032) and 0.7841 (95% CI 0.7638–0.8056) respectively. For QLQ-HCC18 as dichotomized variables, the values were 0.7821 (95%CI 0.7598–0.8043) and 0.7839 (95% CI 0.7621–0.8072) respectively. For HCC18 index score, the values were 0.7791 (95% CI 0.7564–0.8018) and 0.7715 (95% CI 0.7604–0.8034) respectively. All optimisms were within ±0.01 (Table 5). The internally validated optimism-corrected c-index was estimated to be 0.78.

**Table 5:** Performance and internal validation of all the multivariate cox proportional hazard models

MV model	c-index	95% CI	Mean c-index from 1000 bootstraps	95% CI based on 1000 bootstrap samples	Optimism	Optimism in %
M1	0.7872	0.7648–0.8095	0.7891	0.7678–0.8111	0.0019	0.24%
M2	0.7842	0.7617–0.8066	0.7878	0.7660–0.8103	0.0036	0.46%
M3	0.7817	0.7591–0.8043	0.7840	0.7626–0.8066	0.0023	0.29%
M4	0.7810	0.7588–0.8032	0.7841	0.7638–0.8056	0.0031	0.40%
M5	0.7821	0.7598–0.8043	0.7839	0.7621–0.8072	0.0018	0.23%
M6	0.7791	0.7564–0.8018	0.7715	0.7604–0.8034	−0.0076	−0.96%

*MV* multivariate, *CI* confidence interval

M1: the multivariate cox model using QLQ-C30 as continuous variables

M2: the multivariate cox model using QLQ-C30 as dichotomized variables

M3: the multivariate cox model using C30 index score

M4: the multivariate cox model using QLQ-HCC18 as continuous variables

M5: the multivariate cox model using QLQ-HCC18 as dichotomized variables

M6: the multivariate cox model using HCC18 index score

Multiple comparisons showed no statistically significant difference in c-index among related multivariate cox proportional hazard models (Table 6).

**Table 6:** Multiple comparison of c index among various multivariate cox proportional hazard models

MV model	MV model	p-value
M1	M2	0.93067
M1	M3	0.74456
M2	M3	0.81230
M4	M5	0.99435
M4	M6	0.86892
M5	M6	0.87639
M1	M4	0.74687
M2	M5	0.81263
M3	M6	0.87526

*MV* multivariate

M1: the multivariate cox model using QLQ-C30 as continuous variables

M2: the multivariate cox model using QLQ-C30 as dichotomized variables

M3: the multivariate cox model using C30 index score

M4: the multivariate cox model using QLQ-HCC18 as continuous variables

M5: the multivariate cox model using QLQ-HCC18 as dichotomized variables

M6: the multivariate cox model using HCC18 index score

## Discussion

This is the first prospective study to demonstrate that the prognostic significance of QLQ-C30 was not limited to advance-stage HCC patients but valid for newly diagnosed patients with various stages of disease. Worse scores in physical functioning, pain and financial difficulties were associated with shorter OS in dichotomized variable analyses, while worse scores in physical functioning and pain were significant in continuous variable analyses.

This is also the first prospective study to demonstrate that baseline QLQ-HCC18 is a significant prognostication tool for OS in newly diagnosed HCC patients. Worse dichotomized score in fatigue was an independent prognostic factor for shorter OS, while worse continuous scores in fatigue and pain were also significant poor prognostic factors.

Physical functioning domain in the present study concurred with previous findings by Yeo et al. [1] (where physical and role functioning, appetite loss were significant prognostic factors for OS), and Diouf et al. [3] (where physical [dichotomized] or role functioning [continuous] were significant factors).

The HRQOL factors identified in this study varied from previous studies and could be due to a number of reasons. Firstly, patient populations were different, our study involved early as well as advanced stage HCC patients while prior studies involved only advanced stage disease. Secondly, patients of different cultural backgrounds could have different HRQOL perceptions. Thirdly, studies conducted more recently carried more available treatment options than earlier era, which may have led to differences in perception of disease and thus HRQOL. Fourthly, although different studies might utilize the same HRQOL tool, the methodologies of data analysis varied across trials.

The failure to identify consistent HRQOL factors for OS across studies makes clinically meaningful utilization of HRQOL for prognostication difficult. On the other hand, using simple algorithm and calculation, C30 and HCC18 index-scores could be derived from the raw data of all domains and items within QLQ-C30 and QLQ-HCC18 respectively. It is a meaningful representation of the overall HRQOL of an individual patient.

The C30 and HCC18 index-scores were proven to be highly significant prognostic factors for survival, and were more significant than any individual HRQOL factor, whether continuous or dichotomized. When the index-scores were stratified into subgroups, distinct OS outcomes could be identified. Clinical use of either C30 or HCC18 index-score at baseline provides another means of survival estimation in patients with newly diagnosed HCC apart from conventional staging systems. Index-score could be calculated in the clinical setting in a user-friendly manner. With the aid of modern computer technology, patients may be able to self-administer the QLQ-C30 or QLQ-HCC18 questionnaire and have the respective index-score generated by handheld devices.

One limitation of this study was the lack of a separate patient population, for instance, that of a different geographical or cultural background, to allow external validation of the multivariate cox proportional hazard models. However, bootstrapping has enabled internal validation of the multivariate models.

HRQOL assessment is important to aid clinical management in HCC patients. Being an aggressive disease, patients commonly present at advanced stage when treatment option is limited, of modest benefit and associated with disabling toxicities. HRQOL assessment enables the identification of symptoms/problems, whereby symptom control and psychosocial support measures could be offered as part of palliative care in parallel with anti-neoplastic therapy.

HRQOL tools could further be utilized to provide prognostic information. HRQOL analyses may potentially supplement available clinical staging systems in prognostication. External validation of the role of QLQ-C30 and HCC18 index-scores in prognostication in HCC patients is warranted. Index-scoring may prove useful in HRQOL research for other cancer types and further studies are encouraged.

## Conclusions

Both EORTC QLQ-HCC18 and QLQ-C30 measurements at presentation are prognostic for OS in newly diagnosed patients with HCC of various stages. Index-scores of QLQ-HCC18 and QLQ-C30 are highly significant prognostic factors for OS in newly diagnosed HCC patients. Index-scoring provides an effective way to summarize, analyze and interpret raw HRQOL data, and renders QLQ-HCC18 and QLQ-C30 meaningful and communicable in clinical practice. Index-scores of both EORTC QLQ-C30 and QLQ-HCC18 could potentially serve as a standardized tool for future HRQOL research.

## Abbreviations

AFP: α-fetoprotein
BSC: Best supportive care
ECOG: Eastern Cooperative Oncology Group
EORTC: European Organization for Research and Treatment of Cancer
FACT-G: Functional Assessment of Cancer Therapy – General
HBV: Hepatitis B virus
HCC: Hepatocellular carcinoma
HRQOL: Health-related quality-of-life
LEM: Transarterial injection of lipiodol-ethanol mixture
OS: Overall survival
PEI: Percutaneous ethanol injection
RFA: Radiofrequency ablation
TACE: Transarterial chemo-embolisation
